# Epithelial to mesenchymal transition in mammary gland tissue fibrosis and insights into drug therapeutics

**DOI:** 10.7717/peerj.15207

**Published:** 2023-05-09

**Authors:** Mudasir Ahmad Syed, Basharat Bhat, Abiza Wali, Afnan Saleem, Lateef Ahmad Dar, Mudasir Bashir Gugjoo, Shakil Bhat, Sahar Saleem Bhat

**Affiliations:** 1 Division of Animal Biotechnology, Faculty of Veterinary Sciences, Sher-e-Kashmir University of Agricultural Sciences and Technology of Kashmir, India, Srinagar, India; 2 Department of Clinical Biochemistry, University of Kashmir, Srinagar, Jammu and Kashmir, India; 3 Division of Veterinary Surgery, Sher-e-Kashmir University of Agricultural Sciences and Technology of Kashmir, Faculty of Veterinary Sciences and Animal Husbandry, Shuhama, SKUAST-K, India, Srinagar, Jammu and Kashmir, India

**Keywords:** Epithelium-mesenchymal transition, Fibrosis, Goat mammary epithelial cells, MCF10A, Epithelial growth factor, Drug targeting

## Abstract

**Background:**

The epithelial-mesenchymal transition (EMT) is a multi-step morphogenetic process in which epithelial cells lose their epithelial properties and gain mesenchymal characteristics. The process of EMT has been shown to mediate mammary gland fibrosis. Understanding how mesenchymal cells emerge from an epithelial default state will aid in unravelling the mechanisms that control fibrosis and, ultimately, in identifying therapeutic targets to alleviate fibrosis.

**Methods:**

The effects of EGF and high glucose (HG) on EMT in mammary epithelial cells, MCF10A and GMECs, as well as their pathogenic role, were studied. *In-silico* analysis was used to find interacting partners and protein-chemical/drug molecule interactions.

**Results:**

On treatment with EGF and/or HG, qPCR analysis showed a significant increase in the gene expression of EMT markers and downstream signalling genes. The expression of these genes was reduced on treatment with EGF+HG combination in both cell lines. The protein expression of COL1A1 increased as compared to the control in cells treated with EGF or HG alone, but when the cells were treated with EGF and HG together, the protein expression of COL1A1 decreased. ROS levels and cell death increased in cells treated with EGF and HG alone, whereas cells treated with EGF and HG together showed a decrease in ROS production and apoptosis. *In-silico* analysis of protein-protein interactions suggest the possible role of MAPK1, actin alpha 2 (ACTA2), COL1A1, and NF*κ*B1 in regulating TGF*β*1, ubiquitin C (UBC), specificity protein 1 (SP1) and E1A binding protein P300 (EP300). Kyoto Encyclopaedia of Genes and Genomes (KEGG) enrichment suggests advanced glycation end products-receptor for advanced glycation end products (AGE-RAGE) signalling pathway, relaxin signalling pathway and extra cellular matrix (ECM) receptor interactions underlying fibrosis mechanism.

**Conclusion:**

This study demonstrates that EGF and HG induce EMT in mammary epithelial cells and may also have a role in fibrosis.

## Background

Epithelial cells gradually change into mesenchymal-like cells during a process known as epithelial-mesenchymal transition (EMT), losing their epithelial functionality and characteristics (Stone et al., 2016). EMT is characterized by the loss of epithelial cell-to-cell contacts with a decrease in E-cadherin, loss of epithelial cell polarity, an increase in mesenchymal marker expression, Vimentin, and gain of a fibroblastic, motile cell phenotype (Samatov, Tonevitsky & Schumacher, 2013; Stone et al., 2016). EMT has been linked to fibrosis in various organs, including kidney, liver, lungs, intestine, and other organs (Inoue et al., 2015; Lamouille et al., 2013; Rieder et al., 2011). In mammals, EMT is involved in the remodelling of the mammary gland during postnatal development (Nisticò, Bissell & Radisky, 2012). Although EMT has received a great deal of research, it has not yet been fully investigated as a potential therapeutic target for the management and reversal of fibrosis. The process of EMT has taken a centre stage as the convergence point between inflammation and the progression of degenerative fibrotic diseases and cancer (López-Novoa & Nieto, 2009) and is becoming a target of interest for anticancer therapy (Marcucci, Stassi & De Maria, 2016).

Epithelial-mesenchymal transition is executed in response to pleiotropic signalling factors. These factors induce the expression of specific transcription factors called EMT-TFs (*e.g.*, SNAIL, ZEB, TWIST) and miRNAs together with epigenetic and post-translational regulators, many of which are involved in embryonic development, wound healing, fibrosis, and cancer metastasis (Ma et al., 2016).

Fibrosis is characterized by an uncontrolled and excessive deposition of extracellular matrix (ECM) components. Increased ECM deposition evolves into scar tissue and sclerosis which leads to loss of function of the affected organs including, skin, kidneys, lungs, cardiovascular system, liver, pancreas, and intestines (Inoue et al., 2015; Lamouille et al., 2013; Leask & Abraham, 2004; Rieder et al., 2011). Fibrosis also leads to an increase in cytokines, such as TGF*β*1, fibroblast growth factor 2 (FGF2), and platelet-derived growth factor (PDGF) (Xu et al., 2016). Myofibroblasts are the main cell types responsible for the ECM deposition (Gabbiani, 2003). The myofibroblasts, in pathological scenarios, persist and continue to synthesize collagen, leading to fibrotic degeneration. Fibrosis of the mammary tissue causes atrophy and loss of alveolar function with a consequent change in the consistency of the mammary gland and size (Ramos et al., 2020).

Goat is a superior choice to model the human mammary gland when compared to murine and bovine because of similarities in mammary gland size, secretory mechanisms, and morphology with that of humans (Mihevc & Peter, 2013). Long-term mastitis in goats/ruminants can lead to mammary tissue fibrosis which enhances the epithelial-mesenchymal transition by inducing the expression of pro-inflammatory mediators (Liu et al., 2021). Production of inflammatory cytokines, cytotoxic stress in the cells, and DNA damage during tissue fibrosis trigger EMT (Hirasawa et al., 2011; Thiery & Sleeman, 2006). In response to chronic injury or damage, epithelial cells become activated, detach from damaged basement membranes, and begin secreting EMT-promoting cytokines (Zeisberg & Kalluri, 2004). There is a decrease in the expression of epithelial markers such as E-cadherin and zonula occludens-1 (ZO-1). During transition from an epithelial to mesenchymal state, the epithelial cells also show morphology change from polygonal to elongated shape as well as the apical-basal polarity. Transitioned epithelial cells start expressing fibroblast-specific protein (FSP-1), extracellular matrix proteins (*e.g.*, fibronectin, collagen type I and III), and *α*-SMA (Kisseleva & Brenner, 2008). The process of EMT is regulated by SNAIL1/2, ZEB, and TWIST transcriptional factors. Epidermal growth factor (EGF) receptor signalling is implicated in the fibrosis of numerous organs (Hardie et al., 2004; Korfhagen et al., 1994; Wang et al., 2016). In addition, various studies have demonstrated the effect of EGF in inducing EMT (Alipio et al., 2011; Rao, Farley & Pflugfelder, 2010; Wang et al., 2016). EGF is a pleiotropic cytokine that can stimulate proliferation, migration, and adhesion of epithelial cells. Epidermal growth factor (EGF) induces EMT by downregulating E-cadherin *via* E-cadherin internalization, upregulating SNAIL1 and TWIST, and increasing cell motility through Matrix metalloproteinases (MMP)-directed ECM degradation (Ahmed et al., 2006; Lo et al., 2007; Lu et al., 2003).

Hyperglycaemia has also been linked to a pro-fibrotic and inflammatory response, as well as an involvement in EMT (Che et al., 2016; Li et al., 2019a; Li et al., 2019b). High glucose (HG) conditions are reported to stimulate the production of a number of critical fibrogenic cytokines and the process of EMT. Hyperglycaemia has been shown to increase superoxide production, which in turn initiates accelerated advanced glycation end-product (AGE) formation. A study on HG reports induced EMT on retinal pigment cells and shows that AGE-stimulated cells display an altered mesenchymal morphology with a decreased expres”-sion of E-cadherin and an increased expression of vimentin. Also, it elevates the SNAIL mRNA levels (Che et al., 2016). AGEs can directly stimulate the production of ECM by modifying matrix proteins, disrupting matrix-matrix and matrix-cell interactions, and thus contributing to their profibrotic action (Ban & Twigg, 2008).

In the present study, the gene expression of *α*-SMA, Collagen 1, MAPK1, TGF*β*1, and NF-*κ*B was studied to understand the role of epithelial to mesenchymal transition in the pathogenicity of fibrosis.

## Methods

### Cell culture and characterization

MCF10A (ATCC-CRL-10317) cells were purchased and cultured in DMEM-F12 (Sigma-Aldrich, St. Louis, MO, USA) supplemented with hEGF (Himedia, Maharashtra, India), Insulin (Himedia, Maharashtra, India), Hydrocortisone (Himedia, Maharashtra, India), GA-1000 (CCF-3150, Lonza, Walkersville, MD, USA), and cholera toxin (Sigma-Aldrich, St. Louis, MO, USA). GMECs were isolated from whole fresh goat milk obtained from the Mountain Research Centre for Sheep and Goat (MRCSG) of Sher-e-Kashmir University of Agricultural Sciences and Technology, Kashmir (SKUAST-K), Shuhama.

The milk processing protocol has been accepted for patent under Application No. 201911013320 A, Dated: 09/10/2020. The cells were cultured in Dulbecco’s Modified Eagle Medium (DMEM) (4500 mg/l glucose, stable glutamine, sodium pyruvate, and sodium bicarbonate) (Sigma-Aldrich, St. Louis, MO, USA) and supplemented with fetal bovine serum (FBS) (Himedia, Maharashtra, India) and antibiotics including 1 mu/5 ml Penicillin, 1g/ml Gentamicin, 1 g/ml streptomycin, 250 µg/ml Amphotericin B (Himedia, Maharashtra, India).

Cytokeratin 18 (CKT-18) immunofluorescence was used to characterize the GMECs using a specific primary CKT-18 mouse monoclonal antibody (Thermo-Scientific Cytokeratin 18 Antibody (MA5-12104)) and FITC bound anti-mouse IgG secondary immunoglobulin (Fc specific) (F5387 from Sigma-Aldrich, St. Louis, MO, USA). 4′, 6-diamidino-2-phenylindole (DAPI) was used to counterstain the nuclei (Sigma-Aldrich, St. Louis, MO, USA). The images were captured at 100 × using the FLoid cell Imaging workstation (Thermo Scientific).

### Treatment of cells with EGF and HG

GMECs at Passage 2 at a density of 3  × 10^5^ cells were seeded in a 6-well culture plate (BD Falcon) and then cultured at 37 °C and 5% CO_2_ in a water-jacketed humidified incubator (Thermo Scientific) until they reached 80% confluency. MCF10A at a seeding density of 3  × 10^5^ were also seeded in a 6-well culture plate and cultured until they reached 80% confluency. Both the cultures were serum starved for a period of 24 h. After 24 h, the media was replaced with fresh media specific for MCF10A and GMECs supplemented with EGF (600 ng/ml) and/ or HG (6000 mg/l) and a combination of EGF+HG (600 ng/ml + 6000 mg/ml) (Li et al., 2019a; Li et al., 2019b; Xu et al., 2020).

### *In vitro* assessment of morphological EMT-related changes in GMECs and MCF10A

The EMT-related changes in GMECs and MCF10A after treatment were confirmed by visualizing the nuclear and cytoplasmic morphologies. The changes in cell morphologies were assessed under an inverted fluorescent microscope (IX73, Olympus, Tokyo, Japan) after 24 h of treatment with EGF (600 ng/ml) and HG (6000 mg/l) and a combination of EGF+HG (600 ng/ml + 6000 mg/l).

### RNA isolation and cDNA synthesis

Total RNA was extracted using the Trizol method (Invitrogen, Waltham, MA, USA) according to the manufacturer’s instructions. Prior to cDNA synthesis, the quantity and quality of isolated RNA were checked with a UV-visible spectrophotometer at 260 and 280 nm, and the integrity of RNA samples was checked on 1% agarose gel. After RNA extraction, the RNA was treated with DNase using DNase 1 kit (Sigma-Aldrich, St. Louis, MO, USA) to eliminate any genomic DNA contamination. Following that, cDNA synthesis with an equal concentration of RNA (1.5 g/l) in all samples was performed using Thermo Scientific Revert Aid First Strand cDNA Synthesis Kit TM (Lithuania) according to the manufacturer’s protocol. Conventional PCR with cDNA as a template was used to validate cDNA. The PCR primer specifications and annealing temperatures are provided in Tables 1 and 2.

**Table 1:** List of primers used for RT-qPCR for MCF10A cells.

**Gene**	**Forward primer**	**Reverse primer**	**Annealing Temperature** (°C)	**Accession number**
**Cdh1**	5′-GCCTCCTGAAAAGAGAGTGGAAG-3′	5′-TGGCAGTGTCTCTCCAAATCCG-3′	66	GU371438.1
**MAPK1**	5′-GCAACGACCACATCTGCTAC-3′	5′-AGGTTGGAAGGCTTGAGGTC-3′	58	NM_002745.5
**SNAIL1**	5′-TGCCCTCAAGATGCACATCCGA-3′	5′-GGGACAGGAGAAGGGCTTCTC-3′	58	NM_005985
**Collagen1**	5′-ATGGTACTATCTCCTGAGTGCT-3′	5′-TCAGGGCCTGGAAATCCAAC-3′	59	D21337.1
***α*-SMA**	5′-CAGCCAAGCACTGTCAGGAA-3′	5′-TTGTCACACACCAAGGCAGT-3′	60	NM_001613.4
**GAPDH**	5′-GTCTCCTCTGACTTCAACAGCG-3′	5′-ACCACCCTGTTGCTGTAGCCAA-3′	58	NM_001256799
***β*-Actin**	5′-CACCATTGGCAATGAGCGGTTC-3′	5′-AGGTCTTTGCGGATGTCCACGT-3′	64	NM_001101
**Vimentin**	5′-AGGCAAAGCAGGAGTCCACTGA-3′	5′-ATCTGGCGTTCCAGGGACTCAT-3′	60	NM_003380
**TGF *β*1**	5′-TACCTGAACCCGTGTTGCTCTC-3′	5′-GTTGCTGAGGTATCGCCAGGAA-3′	61	NM_000660
**NF *κ*B**	5′-ACACATCCGGACCTCGCA-3′	5′-TCTGAAGCTCTCTCCTCCGC-3′	60	XM_047415742.1

**Table 2:** List of primers used for RT-qPCR for GMECs.

**Gene**	**Forward primer**	**Reverse primer**	**Annealing Temperature** (°C)	**Accession number**
**Cdh1**	5′-TGACACTGACGGTATCAGCG-3′	5′-ATGTGAGCACTTCCGTCTGG-3′	58	XM_005692180.3
**GAPDH**	5′-CGTTCGACAGATAGCCGTAAC-3′	5′-ATGACGAGCTTCCCGTTCTC-3′	58	XM_005680968.3
***β*-Actin**	5′-CCTCTGAACCCCAAAGCCAA-3′	5′-CCACGCTCCGTGAGAATCTT-3′	57	NM_001314342.1
**MAPK1**	5′-GATCTCCGTCGCAGGAAGAC-3′	5′-GCTTTGGAGTCAGCATTCGG-3′	59	XM_018044368.1
**NF *κ*B**	5′-ACACATCCGGACCTCGCA-3′	5′-TCTGAAGCTCTCTCCTCCGC-3′	59	XM_047415742.1
**Vimentin**	5′-CTGAAGCTGCTAACCGCAAC-3′	5′-CATTTCACGCATCTGGCGTT-3′	60	XM_018057155.1
**SNAIL1**	5′-TGTCATGGTGGCACCTGTTT-3′	5′-CCTGTTGGGCCCCGAAAATA-3′	58	XM_013968979.2
**TGF *β*1**	5′-CGGCCAGATTTTGTCCAAGC-3′	5′-GGTCGCGGGTACTGTTGTAA-3′	61	XM_018061650.1
**Collagen1**	5′-CCCAGATGGCTGGTGGATTT-3′	5′-ATACCAGGCTCACCCGTTTG-3′	58	XM_018047868.1
***α*-SMA**	5′-AGCTTTCCAGAACACCACCC-3′	5′-CATTGTCACACACCAAGGCG-3′	55	XM_005698192.3

### Quantitative real-time PCR

Real-Time-qPCR (Light cycler 480 II, RocheTM 480, Germany) was used to determine the gene expression levels of fibrotic and EMT-related genes in MCF10A and GMECs. SYBR Green PCR Master Mix (KAPATM SYBR^®^ qPCR Kit, Kapa Biosystems, Woburn, MA) was used as directed by the manufacturer. The primers used for expression were already reported (E-cadherin, MAPK1, SNAIL1, Collagen 1, *α*-SMA, GAPDH, *β*-Actin, vimentin, TGF*β*1, NF-*κ*B (Kasai et al., 2005; Wang et al., 2016; Xu et al., 2015).

GAPDH and *β*-Actin were used as internal controls for the study. Relative quantification of selected mRNAs was determined using the 2^−ΔΔCT^ method (Livak & Schmittgen, 2001), where ΔΔC_T_ corresponded to the difference between the C_T_ measured for tissue mRNA level and the C_T_ measured for the reference gene mRNA level, ΔC_T_ = C_T_(target gene) − mean of C_T_(*β*-Actin) and C_T_(GAPDH). Experiments were carried out in duplicates.

### Western blotting

Cells were lysed in NP-40 lysis buffer (1% NP-40, 150 mM NaCl, 2 mM EDTA, 20 mM Tris-Cl, 10% glycerol) supplemented with protease and phosphatase inhibitors (50 mM NaF, 1 mM Na3VO4, 1 mM PMSF, 10 ml of 1000X PIC stock/ml of lysis buffer). Cell lysates were centrifuged at 4 °C for 20 min at 10,000 rpm. Bradford assay was used to determine the protein concentration in the supernatants.

The protein samples were denatured by adding 1X Laemmli sample buffer (50 mM Tris-Cl (pH 6.8), 10% glycerol, 2% SDS, 5% b-mercaptoethanol, 0.01% bromophenol blue) followed by boiling at 100 °C for about 5 mins. Equal amounts of protein were loaded onto SDS-PAGE gel and the resolved proteins were electroblotted on PVDF membranes. The blots were then blocked with 5% skim milk or BSA in 1X PBS containing 0.1% Tween-20 (PBS-T) for about 1 hr at room temperature, followed by overnight incubation with specific primary antibodies at 4 °C. Anti-Collagen I antibody (ab260043) from Abcam targeted at the COL1A1 (collagen type 1 alpha1) was used at a dilution of 1:1000. It is a rabbit monoclonal antibody (EPR22894-89) to Collagen I. *β*-actin was used as the loading control. *β*-actin antibody from Cell signalling technology (4967S) was used at a dilution of 1:1000 Afterward, blots were washed three times with chilled 1X TBS containing 0.05−0.1% Tween-20 (TBS-T) and incubated with secondary antibody (DyLight 800, A32732) at 1:20000 dilution for 1–2 h. The blots were again washed three times with 1X TBS-T, followed by infrared detection using the Li-Cor Odyssey imaging system (Ali et al., 2021; Bhat et al., 2020).

### ROS generation assay

Amplex red catalase assay kit was used for the extracellular H_2_O_2_ quantification in the cell culture media as per the manufacturer‘s protocol. Briefly, cells plated in 6-well plates at a density of ∼5 ×10^5^ cells per well were treated with the appropriate concentration of EGF, HG, or EGF+HG. 50 µl of Amplex Red reagent/horseradish peroxide (HRP) working solution (50 µl of 10 mM Amplex Red reagent, 100 µl of 10 U/ml HRP stock solution, and 4.85 ml of 1X reaction buffer) was added to each well containing treated and control samples and incubated in dark for 30 min in a CO_2_ incubator. The fluorescence signal was determined using a spectrofluorophotometer (RF-5301; Shimadzu, Kyoto, Japan) with an excitation wavelength of 568 nm and an emission wavelength of 581 nm (Ali et al., 2021; Bhat et al., 2020).

### Quantification of apoptosis

Cell death detection ELISA kit (Roche Molecular Biochemicals, Indianapolis, IN) was used to detect apoptosis after EGF, HG, and EGF/HG treatment to GMECs and MCF10A. This assay is based on a quantitative sandwich ELISA using antibodies directed against DNA and histones to detect mono and oligonucleosomes in the cytoplasm of cells undergoing apoptosis. GMECs and MCF10A cells were grown till they were 80% confluent. The cell medium was replaced with serum-free medium after 24 hr. The cells were serum starved for 12 h. Cells were counted on a haemocytometer prior to plating on a 96-well plate & each well has around 200 cells per well. This was followed by treatment with EGF, HG, and EGF/HG together for 6 h. After 6 h the plate was centrifuged and the supernatant was removed. The ELISA was then carried out according to the manufacturer’s protocol and as performed previously (Bhat et al., 2016).

### Network analysis

To identify the functional role of the differentially expressed genes and their interacting partners, genes were subjected to the STRING database and ENCODE database for possible protein-protein and protein-transcription factor interactions, respectively. To identify the possible protein-drug and protein-chemicals interaction, Comparative Toxicogenomics Database (CTD) (Version Nov. 2016) and the Drug Bank database (Version 5.0) respectively was used. KEGG-KASS servers were used to elucidate the functional role of differentially expressed genes.

### Statistical analysis

The data obtained is represented as mean ± S.E. and *p* < 0.05 was considered significant. Assays were performed in triplicate and repeated at least three times independently for each cell line. Data was analysed using two-way analysis of variance (ANOVA) and Dunnett’s multiple comparison post hoc tests. Two-way ANOVA indicated there was a significant difference overall for EGF+HG treatment (*p* < 0.05) and the post-test indicated that there were differences against each control ( *p* < 0.05) as displayed in the graph.

## Results

### GMECs express epithelial cells specific marker protein CKT-18

**Figure 1:**
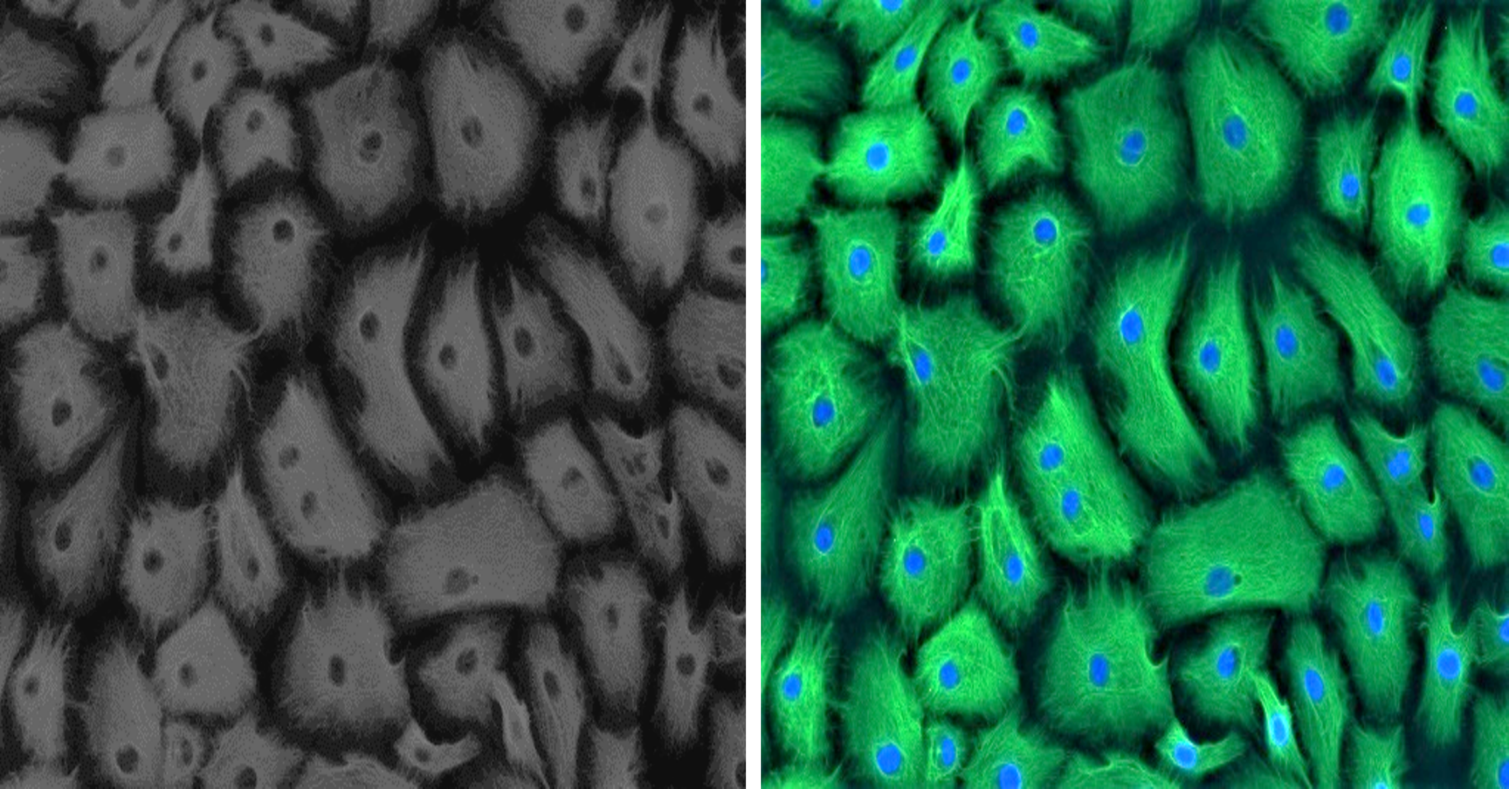
Positive immunofluorescence for epithelial cell marker Cytokeratin 18 on GMECs.

Established GMECs cell culture was phenotypically assessed using a marker for epithelial cells to ascertain its purity. Epithelial characteristics of established GMECs were examined for intermediate filament protein CKT-18 at an early passage (P1). Immunostaining results show that the established cultures are positive for a significantly expressed green-colored CKT-18 (Fig. 1), thus confirming their epithelial nature (the nuclei appear blue in color).

### Effect of EGF, HG and EGF+HG treatment on the morphology of GMECs and MCF10A

Morphological changes from epithelial to mesenchymal after treatment of GMECs and MCF10A cells with 600 ng/ml of EGF and 6000 mg/ml high glucose for 24 h were evident as visualized under an inverted light microscope. Control GMECs and MCF10A cells showed cobblestone and cuboidal shape respectively. But after treatment, GMECs and MCF10A cells attained flat, elongated, spindle-shape features. However, both the cell lines when treated with EGF+HG (600 ng/ml + 6000 mg/l), showed no changes in morphology, indicating that combination treatment may not stimulate EMT-related phenotypic changes in mammary epithelial cells (Supplementary File).

#### Characterization of EMT in GMECs and MCF10A cells

**Figure 2:**
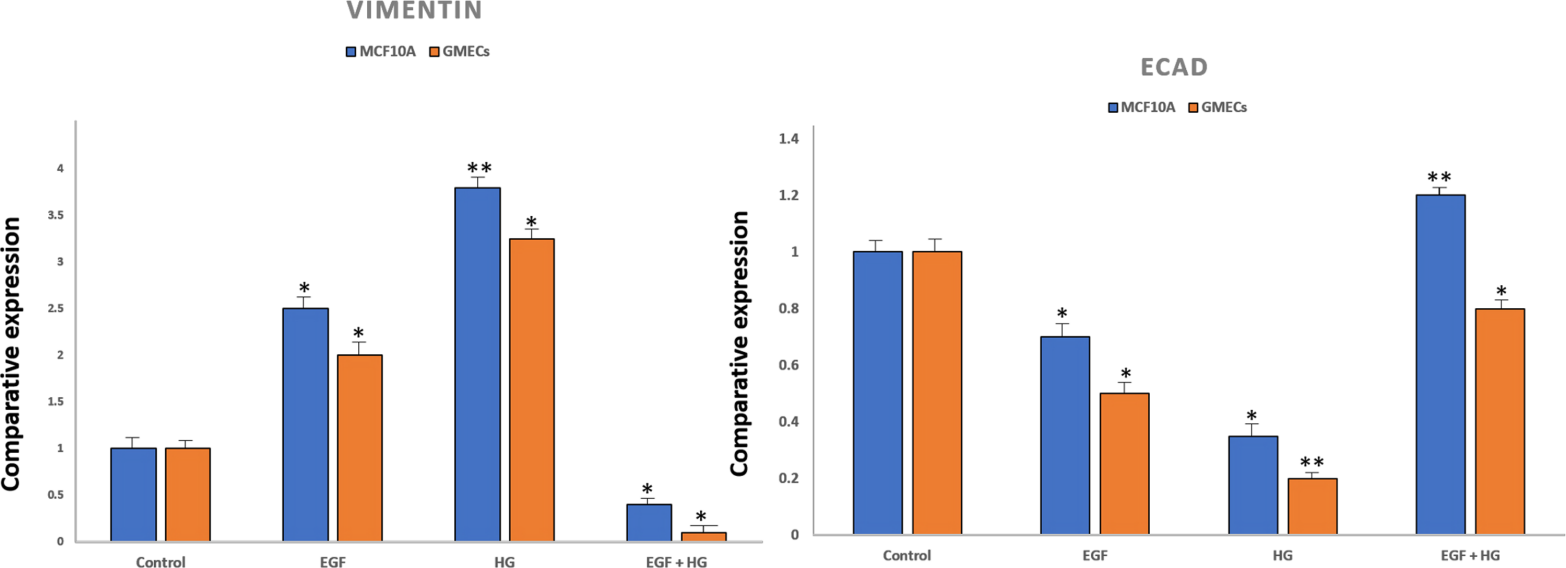
Expression of epithelial markers after treatment of cells with EGF and HG. The expression of E-cadherin significantly reduced in MCF10A as well as GMEC cells upon exposure to EGF or HG, whereas the expression of Vimentin increased upon treatment with EGF and HG, but decreased when treated with EGF and HG together in both MCF10A and GMEC cells. Data are mean ± S.E . *p* values determined by two-way ANNOVA followed by Dunnett’s multiple comparisons post hoc test (* = *p* value 0.001, ** = *p* value 0.0001).

To confirm EMT, cells were characterized by analysing the gene expression of cell surface markers *i.e.,* vimentin and E-cadherin by qPCR. After treatment with EGF and HG, there was a marked increase in the expression of vimentin and reduced expression of E-cadherin in GMECs and MCF10A cells. However, the treatment of cells with EGF+HG reduced the gene expression of vimentin with no changes in E-cadherin gene expression (Fig. 2).

### EGF and HG induces EMT in mammary epithelial cells and upregulates the fibrogenic markers

**Figure 3:**
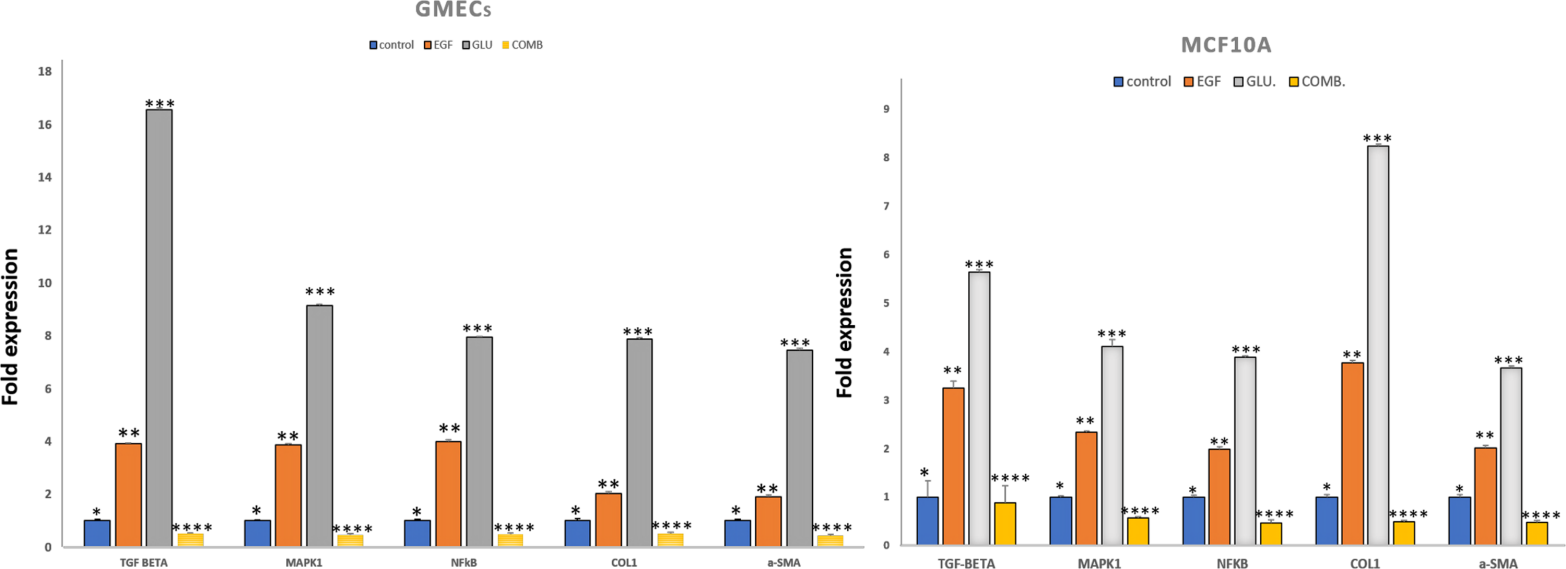
Exposure of mammary epithelial cells to HG and EGF increased the expression of fibrotic markers & signalling molecules. The expression of the markers was significantly increased in GMECs & MCF-10A cells upon exposure to HG and EGF. Data are mean ± SEM. *p* values determined by two-way ANNOVA followed by multiple comparisons test (* = *p* value 0.01, ** = *p* value 0.001, *** = *p* value 0.0001).

We investigated the effects of HG and EGF on EMT in GMECs and MCF10A by evaluating the gene expression of Vimentin, SNAIL1, and E-cadherin through qPCR analysis. Gene expression of SNAIL1, a classical transcriptional factor in EMT and fibrosis and a suppressor of E-cadherin was upregulated in the HG and EGF groups *vs* nontreated groups in GMECs and MCF10A cells. The gene expression of E-cadherin was downregulated in treated groups as compared to control groups in GMECs and MCF10A cells. The gene expression of vimentin was upregulated in the HG and EGF groups when compared to control groups in both GMECs and MCF10A cells. MCF10A treated with EGF demonstrated a significant increase of 2.029-fold (*p* = 0.041) gene expression of *α*-SMA (myofibroblast marker) and collagen1 which is the main component of extracellular matrix proteins also showed a significant increase of 3.775-fold expression when compared to control cells (*p* = 0.048) (Fig. 3). Similarly, MCF10A cells when treated with HG showed 3.678 (*p* = 0.039) and 8.243 (*p* = 0.045)-fold increase in *α*-SMA and collagen1 expression respectively when compared to control cells.

In GMECs treated with EGF, qPCR analysis showed 1.918 (*p* = 0.057) and 2.027-fold (*p* = 0.073) increase in the gene expression of *α*-SMA and collagen1 respectively when compared to control (*P* < 0.05). But with HG treatment, *α*-SMA and collagen1 both increased up to 7.464 (*p* = 0.065) and 7.889 (*p* = 0.040)-fold gene expression in GMECs when compared to control cells (*P* < 0.05). Furthermore, the gene expression of the said markers in GMECs and MCF10A cells treated with EGF+HG was found to be less when compared to the ones treated with EGF and HG individually. In MCF10A, there was a significant decrease of 0.488 (*p* = 0.043) and 0.496 (*p* = 0.032) fold in the gene expression of *α*-SMA and collagen1 respectively by EGF+HG. Similarly, in GMECs, the gene expression of *α*-SMA and collagen1 decreased significantly up to 0.435 (*p* = 0.056) and 0.517 (*p* = 0.054) fold respectively.

**Figure 4:**
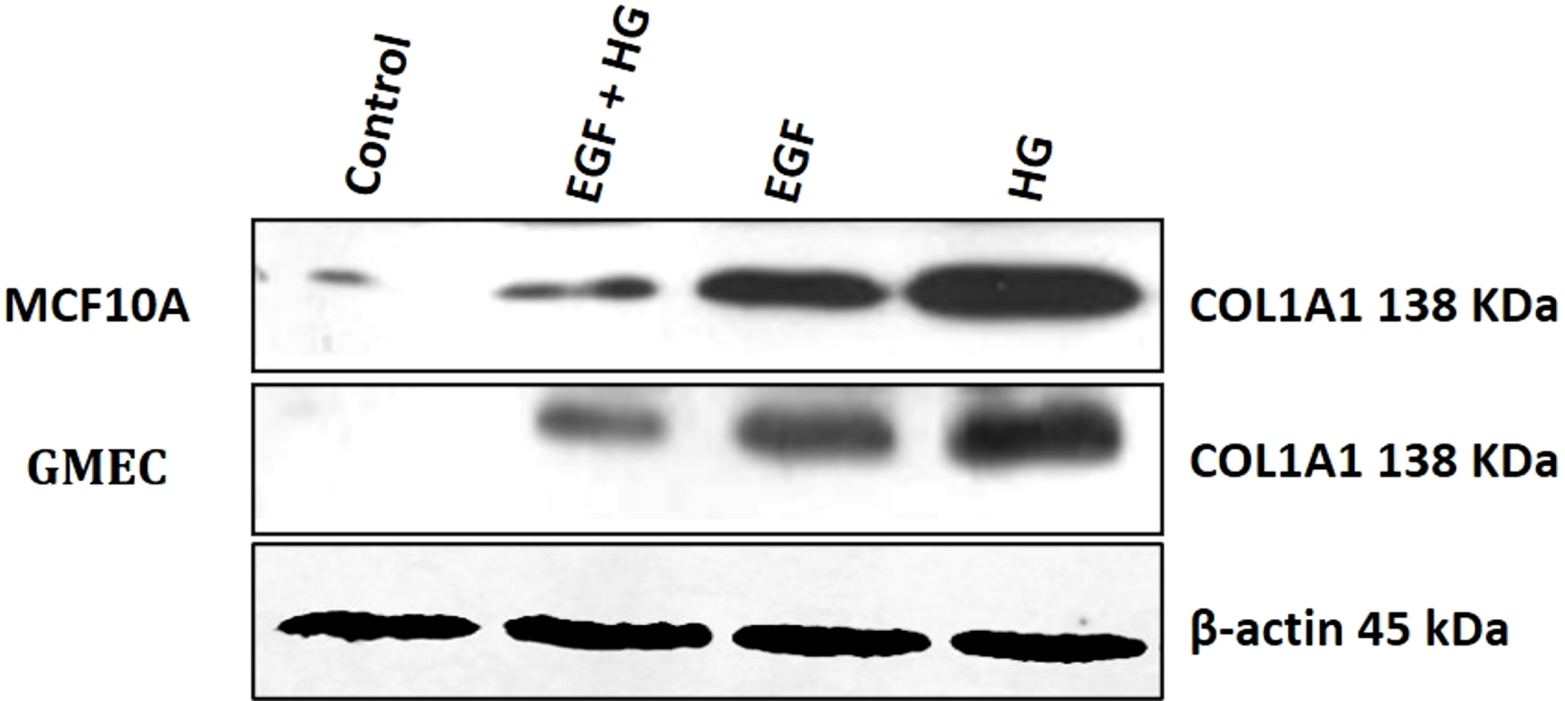
Treatment of GMEC and MCF-10A cells with EGF, HG and EGF +HG increases collagen 1 expression. The protein expression of collagen1 was examined in GMEC and MCF-10A cells before and after treatment with EGF, HG and EGF +HG. Protein expression of collagen1 increased in response to the treatment and the highest expression was observed in the cells treated with HG. *β*-actin was used as a loading control.

Since collagen1 is the main component of the extracellular matrix proteins, we examined the effect of EGF and HG treatment on the protein expression of collagen1. We observed that the protein expression of collagen1 increases in cells when treated with HG and EGF, with the highest expression in cells treated with HG, but the protein expression of collagen1 is decreased in cells treated with EGF+HG when compared to the cells treated with EGF and HG alone (Fig. 4).

### EGF and HG upregulates the expression of TGF-*β* and NF-*κ*B

The gene expression of TGF-*β* and NF-*κ*B were evaluated in GMECs and MCF10A treated with EGF or HG and EGF+HG. The gene expression of TGF-*β* and NF-*κ*B in both cell lines was upregulated. qPCR analysis revealed 1.995 (*p* = 0.053) and 3.254 (*p* = 0.136)-fold increase in the gene expression of NF-*κ*B and TGF-*β* in MCF10A respectively by EGF treatment when compared to control cells. Similarly, HG treatment in MCF10A showed 3.892 (*p* = 0.026) and 5.643 (*p* = 0.054)-fold increase in NF-*κ*B and TGF-*β* respectively when compared to control cells.

In GMECs, treatment with EGF, NF-*κ*B and TGF-*β* gene expression was increased by 4 (*p* = 0.063) and 3.917 (*p* = 0.039) fold respectively when compared to control. Similarly, HG treatment caused up to 7.944 (*p* = 0.026) and 16 (0.065)-fold increase in the gene expression of NF-*κ*B and TGF-*β* respectively when compared to control cells. MCF10A and GMECs when treated with EGF+HG combination, the gene expression of said markers decreased significantly when compared to the ones treated with EGF and HG individually. In MCF10A, qPCR analysis showed a significant decrease of 0.478 (*p* = 0.058) and 0.886 (*p* = 0.347) fold in the gene expression of NF-*κ*B and TGF-*β* respectively when compared to EGF and/or HG treated groups (*P* < 0.05). Similarly, in GMECs, qPCR revealed a significant decrease of 0.4829 (*p* = 0.050) and 0.5 (*p* = 0.040) fold in the gene expression of NF-*κ*B and TGF-*β*1 respectively when compared to EGF and/or HG treated groups (Fig. 3).

### EGF and HG activates MAPK1 in GMECs and MCF10A cells

On treatment with EGF and HG, the gene expression of MAPK1, a serine/threonine kinase was evaluated for MCF10A and GMECs. qPCR analysis showed a 2.345 (*p* = 0.029) and 4.112-fold (*p* = 0.138) increase in the gene expression of MAPK1 in MCF10A by EGF and HG respectively when compared to control. GMECs treated with EGF and HG showed a 3.877 (*p* = 0.0435) and 9.152-fold (*p* = 0.054) increase in the gene expression of MAPK1 when compared to control cells. However, for both GMECs and MCF10A cells, the gene expression of MAPK1 was reduced when treated with EGF+HG. In MCF10A, qPCR analysis showed a 0.57-fold (*p* = 0.030) decrease in the gene expression of MAPK1 by EGF+HG when compared to EGF and /or HG treated groups. qPCR analysis of gMECs showed 0.468-fold (*p* = 0.055) decrease in the gene expression of MAPK1 when compared to EGF and/or HG treated groups (Fig. 3).

### EGF and HG treatment leads to ROS production and induction of apoptosis in GMECs and MCF10A cells

**Figure 5:**
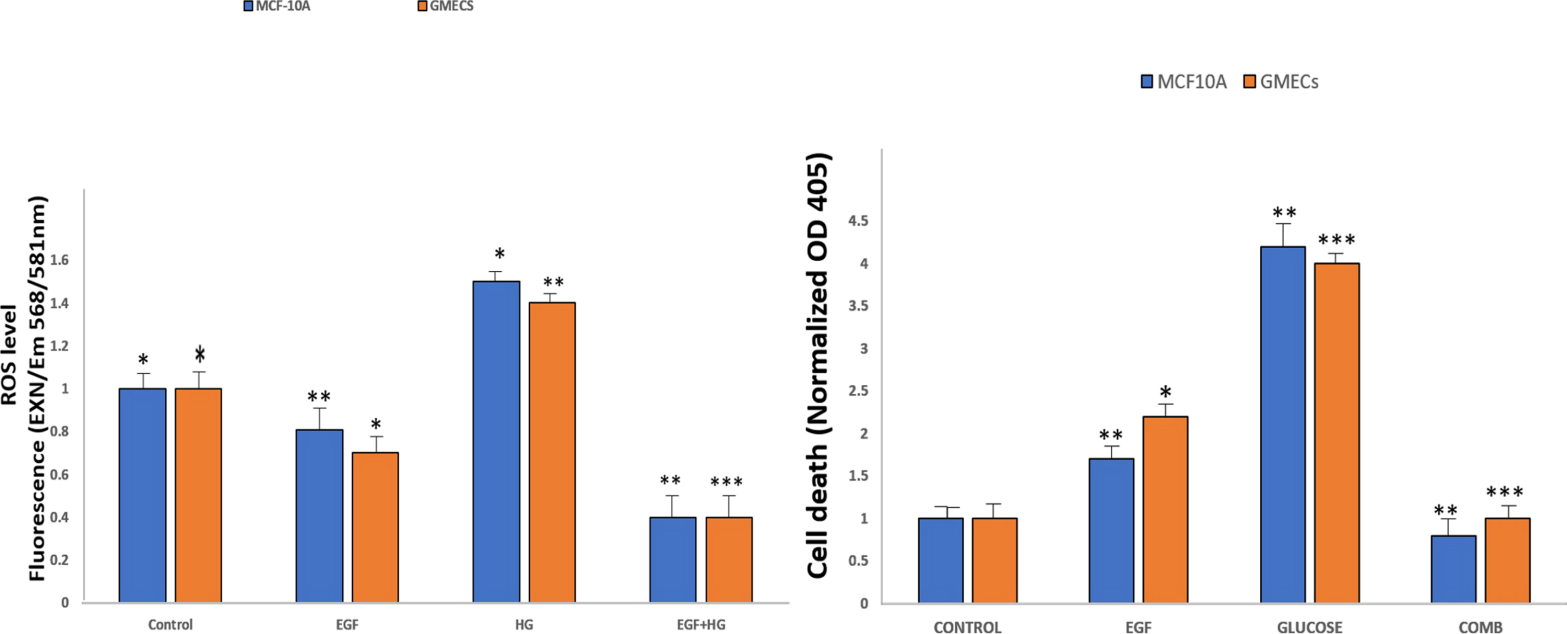
EGF and HG treatment leads to ROS production and induction of apoptosis in GMECs and MCF10A cells. The ROS production as well as apoptosis was highest in the cells treated with HG, with the lowest levels of both observed in cells treated with EGF and HG together. Data are mean ± SEM. *p* values determined by two-way ANNOVA followed by multiple comparisons test (* = *p* value0.01, ** = *p* value 0.001, *** = *p* value 0.0001).

Amplex Red Assays were used to evaluate the effects of EGF and HG treatment on the production of ROS in GMECs and MCF10A. Results showed that ROS generation significantly increased in cells treated with EGF and HG when compared to control, with the highest ROS levels seen in cells treated with HG. Interestingly, ROS levels decreased when the cells were treated with EGF and HG together (Fig. 5A). Increased oxidative stress has been reported in diabetic complications, which further has been shown to lead to diabetes-related vascular calcification.

Cells were treated with EGF and HG as well as EGF in combination with HG in order to examine the impact of this increased ROS production on apoptosis in GMECs and MCF10A cells. Treatment of cells with EGF and HG lead to an increase in apoptosis, with the highest cell death being seen in cells treated with HG. Cells treated with EGF alone also showed an increase in cell death, but it was lower than what we observed in cells treated with HG. Cells treated with EGF and HG together showed a marked decrease in apoptosis when compared to the control (Fig. 5B). This suggests that EGF and HG may play a role in ROS generation and, as a result of increased levels of oxidative species, cell apoptosis.

### Network analysis

#### Protein-protein interaction network

**Figure 6:**
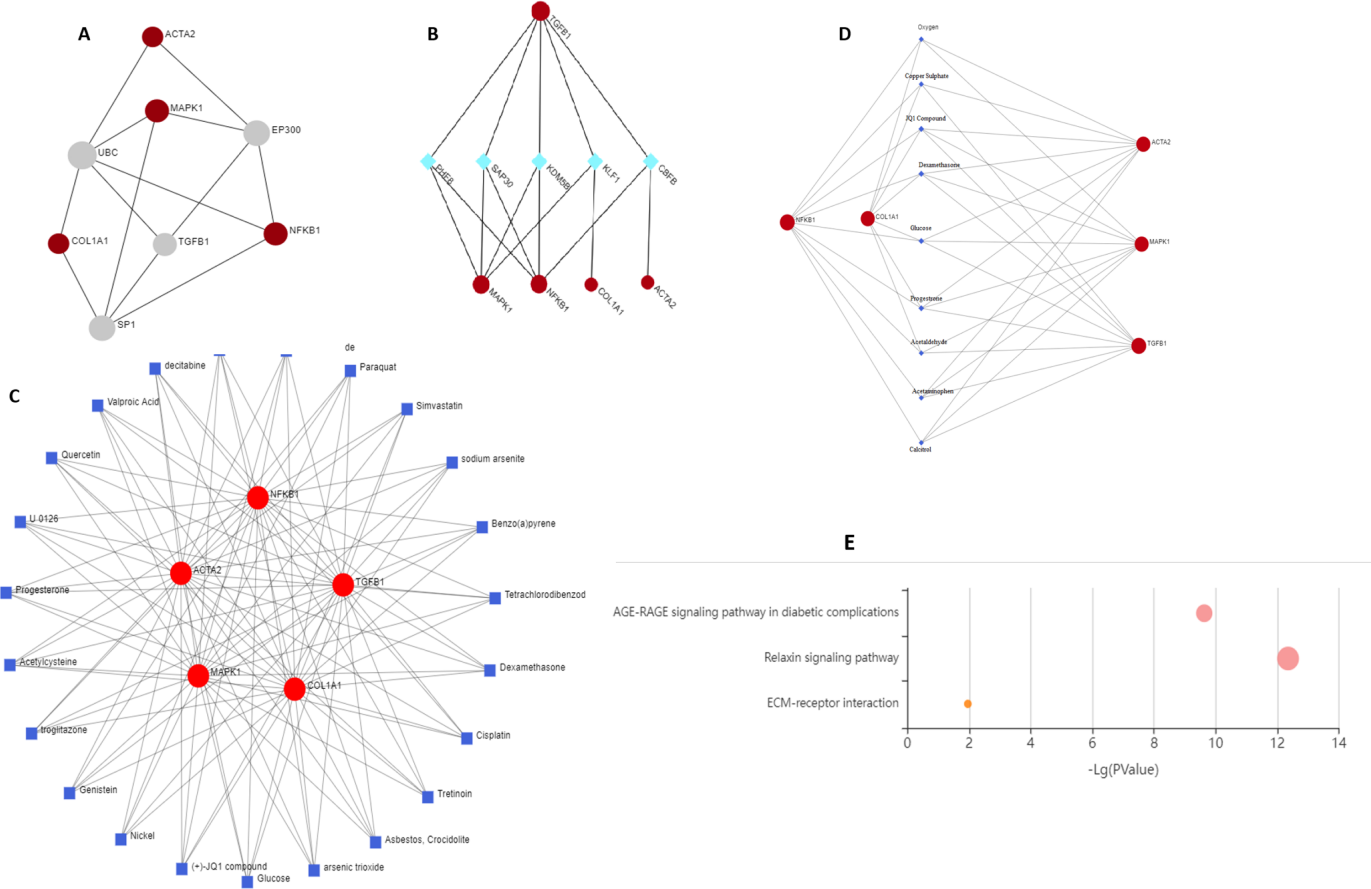
Schematic diagram of network and pathway analysis of compound–protein interactions. (A) Protein-protein interaction using STRING database, (B) protein-transcription factor interactions using ENCODE database, (C and D) protein-chemicals and protein-drug interaction using Comparative Toxicogenomics Database (CTD) (Version Nov. 2016) and DrugBank database (Version 5.0) and (E) KEGG pathways analyzing using KEGG-KASS server.

Two topological features, *Betweenness in nodes (>5)* and *Degree (>5)* were calculated to identify key nodes. Higher the two quantitative values of a gene, the more important it is in the PPI network (Fig. 6A). Our analysis suggests the possible role of MAPK1, ACTA2, COL1A1, and NF-*κ*B1 in regulating TGF*β*1, UBC, SP1, and EP300. TGF*β*1 has a known role in cystic fibrosis. Members of the collagen gene family act as a target of fibrotic diseases regulated by gene expression of EP300. Blocking SP1 inhibits extracellular matrix gene expression *in vitro* and *in vivo* for the treatment of tissue fibrosis. We identified UBC interacts directly with five key genes associated with fibrosis, which indicates a possible role of this gene. Further study is required to validate the possible role of UBC in fibrosis.

#### Protein-TF network

KDM5B (Lysine Demethylase 5B) is a histone demethylase that demethylates ’Lys-4’ of histone H3, thus playing a central role in histone code. It acts as a transcriptional corepressor for FOXG1B and PAX9. It favours the proliferation of breast cancer cells by repressing tumour suppressor genes such as BRCA1 and HOXA5. CBFB (Core-binding factor subunit beta) gene encodes a transcription factor that has emerged as a highly mutated driver in a variety of human cancers including breast cancer. CBFB binds to mRNAs through hnRNPK and facilitates translation initiation by eIF4B. *RUNX1* mRNA encoding the transcriptional partner of CBFB is bound and translationally regulated by CBFB. The nuclear CBFB/RUNX1 complex transcriptionally represses the oncogenic NOTCH signalling pathway in breast cancer. Breast cancer cells avoid translation and transcriptional surveillance by downregulating CBFB (Malik et al., 2019) (Fig. 6B).

#### Protein-chemical and drug network

This network enables us to get a quick overview of the interaction of the chemical with its interaction partners. Fibrosis is characterized by accumulation of excessive connective tissue in the extracellular matrix and its causes include resveratrol (Zhang et al., 2015), paraquat (Li et al., 2017), simvastatin (Yang, Chen & Sun, 2013), sodium arsenite (Jiao et al., 2019), asbestos, crocidolite (Tamminen et al., 2012), nickel (Wu et al., 2012), acetylcysteine (Fen et al., 2019), quercetin (Sellarés & Rojas, 2019), arsenic trioxide (Luo et al., 2014) for pulmonary fibrosis, liver fibrosis caused by tetrachlordibenzodioxin (Fling et al., 2020), (+) JQ1 compound (Li et al., 2020), ganister (Arora et al., 2016) and renal fibrosis by cisplatin (Sharp & Siskind, 2017). Drugs reported to cause fibrosis include dexamethasone (Li et al., 2019a; Li et al., 2019b), glucose (Talakatta et al., 2018), acetaminophen (Bai et al., 2017), calcitrol (Martínez-Arias et al., 2021), progesterone (Vafashoar et al., 2021) (Figs. 6C and Fig. 6D).

#### KEGG enrichment

Genes were mapped to the KEGG database to gain biological insights and identify potential pathways dysregulated by the genes under study. Three critical pathways ECM receptor interaction, AGE-RAGE, and relaxin signalling were discovered to be significantly impacted (Fig. 6E).

Diabetic heart fibrosis is mediated by RAP1A activation of AGE-RAGE signalling (Zhao, Randive & Stewart, 2014). Relaxin reduces pathological collagen production by blocking its synthesis and secretion from myofibroblasts (Samuel, 2005). Relaxin increases the expression and activity of MMPs while decreasing the levels of their natural inhibitors, tissue inhibitors of metalloproteinases) to aid in the breakdown of abnormal collagen build up. Relaxin has a crucial role in the progression and therapy of fibrosis, according to extensive *in vitro* and *in vivo* evidence. New research suggests that other relaxin family peptides, such as relaxin-3, may play a role in fibrosis (Benjamin, 2001). Fibrosis progression involves both ECM-driven and cell-intrinsic/autonomous mechanisms. ECM is a major source of biochemical and biomechanical signals which are transduced and integrated to determine tissue organization and function. Excessive ECM production and turnover characterize organ fibrosis. Dysregulation of ECM remodelling enzymes causes ECM structure disintegration and fragmentation which contributes to fibrosis progression (Erikson & Unemori, 2001). A comprehensive understanding of the spatial and temporal alterations in ECM composition will elucidate the mechanism underlying the progression of organ fibrosis.

## Discussion

Epithelial-mesenchymal transition (EMT) marks the gradual transition of epithelial function and features of epithelial cells into mesenchymal-like cells. The epithelial-mesenchymal transition plays a role in the fibrosis of numerous organs including kidneys, lungs, and liver (Carew, Wang & Kantharidis, 2012; Chapman, 2011; Lee, Kim & Park, 2014; Nowrin et al., 2014).

Role of EGF and HG in inducing EMT in normal immortalized breast epithelial cells of humans, MCF10A, and milk-derived GMECs and their involvement in fibrosis were assessed in this study. MCF10A and GMECs were treated with EGF and HG and a change in their morphology from cobblestone and cuboidal to elongated and spindle shape respectively was observed. Cells showed an increase in the expression of EMT markers vimentin and SNAIL1, with decreased expression of E-cadherin. The loss of E-Cadherin disrupts the epithelial integrity and promotes EMT. Gene expression of *α*-SMA and collagen1 showed a significant increase in both MCF10A and GMECs. *α*-SMA has been shown to promote EMT in embryogenesis and aids in wound healing in normal epithelial cells. *α*-SMA is also implicated in the progression and metastasis of carcinomas. The effects of EGF and HG treatment on the COL1A1 protein expression levels in GMECs and MCF10A cells was investigated since collagen1 is the most abundant extracellular protein and has been extensively implicated in fibrosis. COL1A1 protein expression increased in cells treated with EGF and further increased in cells treated with HG. However, when these cell lines were treated with both EGF and HG, the protein expression of COL1A1 is significantly reduced. Collagen is an important fibrosis regulator and has been shown that fibrosis occurs when the rate of synthesis of new collagen is more than the rate of its degradation.

*α*-SMA expression correlates with the activation of myofibroblasts (which arise from EMT in this case) plays an important role in fibrogenesis. Myofibroblasts, in an activated state, cease to proliferate and start to synthesize large amounts of extracellular component proteins; collagen1 being one of them (Cherng, Young & Ma, 2008). *In-vitro* induction of EMT by TGF-*β*/EGF in murine-induced pluripotent stem cell-derived alveolar Type II-like cells has been previously reported (Alipio et al., 2011).

For HG-treated cells, upregulated expression of vimentin and SNAIL1 with decreased expression of E-cadherin when compared to control was observed. A significant increase in the gene expression of *α*-SMA and collagen1 fibrotic markers was observed in both the cell lines. The results are supported by a study on retinal pigment epithelial cells, which demonstrated that HG is able to induce EMT as well as upregulate fibrotic markers (Che et al., 2016).

The gene expression of signalling molecules NF-*κ*B, TGF-*β*, and MAPK1 was evaluated. EGF and HG are involved in activating TGF*β via* NF-*κ*B pathway (Suryavanshi & Kulkarni, 2017; Wang et al., 2016). NF-*κ*B mediates EMT and is also involved in fibrosis (López-Novoa & Nieto, 2009). It acts as a central mediator in the induction of TGF*β* in monocytes from patients with idiopathic myelofibrosis (Rameshwar et al., 2000). A major EMT inducer TGF*β* is endogenously expressed in injured tissues as well as in patients suffering from fibrotic diseases (Alipio et al., 2011). It has been shown that *in vitro* supplementation of TGF-*β* causes EMT in bovine mammary epithelial cells (Chen et al., 2017). The gene expression analysis of NF-*κ*B and TGF-*β* in MCF10A and GMECs showed a significant increase in the expression of these genes when compared to control groups.

The expression of MAPK1 was significantly elevated in both the EGF and HG-treated cells. Fibrotic marker MAPK1 controls cellular processes including cell growth, proliferation, migration, protection from apoptosis, and myofibroblast transformation (Madala et al., 2012). MAPK has been shown to regulate various cellular processes involved in fibrosis, like cell growth, cellular migration, protection of the cells from apoptosis, in addition to myofibroblast transformation. MAPK pathway is also known to mediate EMT in alveolar epithelial cells, showing the involvement of MAPK in EMT (Huang et al., 2016).

The gene expression of the evaluated markers by EGF is less when compared to HG. As a result, it was concluded that HG is a stronger inducer of EMT than EGF, though this needs to be investigated further. The combined effect of EGF and HG was investigated in MCF10A and GMECs, which showed the opposite effects as EGF and HG alone. The cells which were treated with EGF+HG showed decreased expression of EMT markers as well as signalling molecules indicating that EGF in combination with HG does not induce EMT in both the cell lines and consequently, it may not activate fibrosis as well. It could be because EGF is known to lower plasma glucose levels, according to a study on diabetic mice (Lee et al., 2008) and may, therefore, counteract the effects of HG in mammary epithelial cells, when both EGF and HG are used. This counteraction of HG will not happen in cells when they are treated with HG alone and this is why decreased expression of the EMT markers was observed. Further elucidation on the combined role of EGF and HG on EMT and fibrosis needs to be done.

The effects of EGF and HG treatment on ROS and apoptosis in both cell lines were investigated. ROS levels and cell death were the highest in cells treated with HG. ROS production and apoptosis increased in cells treated with EGF+HG alone when compared to the control. Furthermore, ROS levels and cell death were decreased when the cells were treated with EGF in combination with HG. Increased ROS levels have been implicated in the onset and progression of fibrosis.

Protein-protein interactions are essential for understanding cell physiology in normal and diseased states. Protein-protein interaction network is a mathematical representation of the physical contacts between proteins in the cells. Our analysis suggests the possible role of MAPK1, ACTA2, COL1A1, and NF- *κ*B1 in regulating TGF*β*1, UBC, SP1, and EP300. The characterization of drug-protein interaction networks with biological characteristics are involved in various features of drugs and target proteins (*e.g.*, chemical substructures, pharmacophores, functional sites, and pathways) and complicated associations between the heterogeneous features. As reported earlier, fibrosis is characterized by the accumulation of excessive connective tissue in the extracellular matrix and its causes include resveratrol (Zhang et al., 2015), paraquat (Li et al., 2017), simvastatin (Yang, Chen & Sun, 2013), for pulmonary fibrosis, tetra-chlordibenzodioxin (Fling et al., 2020), (+) JQ1 compound (Li et al., 2020), genistein (Arora et al., 2016) causes liver fibrosis and cisplatin (Sharp & Siskind, 2017) causes renal fibrosis. Drugs reported to cause fibrosis include dexamethasone (Li et al., 2019a; Li et al., 2019b), glucose (Talakatta et al., 2018), acetaminophen (Bai et al., 2017), calcitriol (Martínez-Arias et al., 2021), and progesterone (Vafashoar et al., 2021). Functional domains, pathway modules, and biological pathways were obtained from KEGG databases. In diabetic complications, the AGE-RAGE signalling pathway has been linked to increased oxidative stress and diabetes-related vascular calcification (Zhao, Randive & Stewart, 2014). *In vitro* and *in vivo* evidence reveals a crucial role of relaxin in the progression and therapy of fibrosis. Research suggests that other relaxin family peptides, such as relaxin-3, may also play a role in fibrosis (Benjamin, 2001). An inclusive understanding of the temporal and spatial alterations in ECM composition will further elucidate the progression of the organ fibrosis mechanism. Dysregulation of ECM remodelling enzymes causes ECM structure disintegration and fragmentation, ultimately contributing to fibrosis progression (Erikson & Unemori, 2001). These protein-protein interaction networks, drug-protein interaction networks, and biological pathways consisting of associations will help in extracting meaningful drug-protein interaction signatures. This research opens the door to using EMT to target and treat fibrosis, but more research is required before EMT can be effectively used to treat and reverse fibrosis of the mammary gland.

## Conclusion

This study demonstrates that EGF and HG induces EMT in mammary epithelial cells and are also involved in the pathogenicity of fibrosis. Finding new targets for the treatment of mammalian fibrosis can be aided by understanding the mechanism of EGF and HG-induced EMT in mammary epithelial cells. Potential applications for EMT include the treatment of fibrosis and its reversal.

##  Supplemental Information

10.7717/peerj.15207/supp-1 Supplemental Information 1 HG and EGF induced morphological change of MCF-10A and GMECs

10.7717/peerj.15207/supp-2 Supplemental Information 2 Unedited Western blots

10.7717/peerj.15207/supp-3 Data S1 Raw data

## List of abbreviations

EGF: Epidermal growth factor
HG: High Glucose
GMECs: Goat mammary epithelial cells
EMT: Epithelium-mesenchymal transition
ECM: Extracellular matrix
CKT-18: Cytokeratin 18
*α*-SMA: *α*-smooth muscle actin
MMP: Matrix metalloproteinase
TGF*β*1: Transforming growth factor-beta 1
MAPK1: Mitogen activated protein kinase 1
NF *κ*B: Nuclear factor kappa B
DMEM: Dulbecco’s Modified Eagle Medium
FBS: Fetal bovine serum
CBFB: Core-binding factor subunit beta
AGE: Advanced glycation end-product

## Additional Information and Declarations

### Data Availability

The following information was supplied regarding data availability: The raw data is available in the Supplemental Files.
