# Supplementary material for: Epithelial to mesenchymal transition in mammary gland tissue fibrosis and insights into drug therapeutics
Source: PeerJ. 2023 May 9;11:e15207. doi: 10.7717/peerj.15207 (PMC10178283; doi:10.7717/peerj.15207)
Supplement: Supplemental Information 1 [file peerj-11-15207-s001.docx]

**MCF-10A**


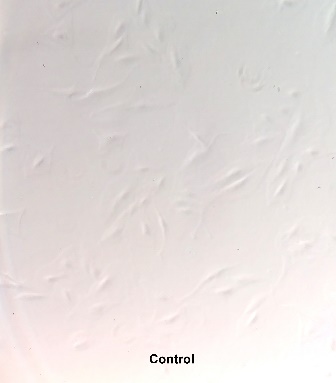

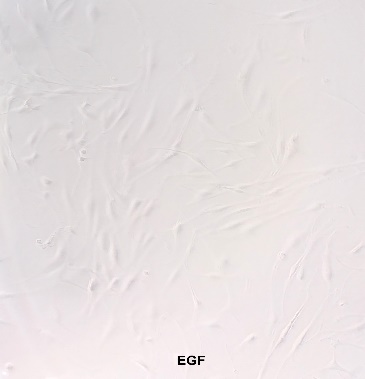

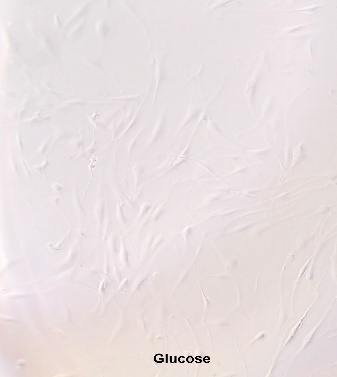

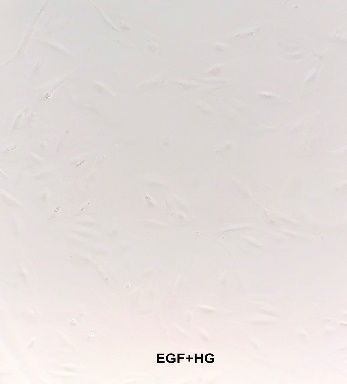


**GMECS**


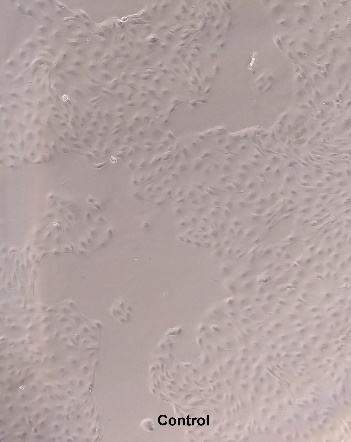

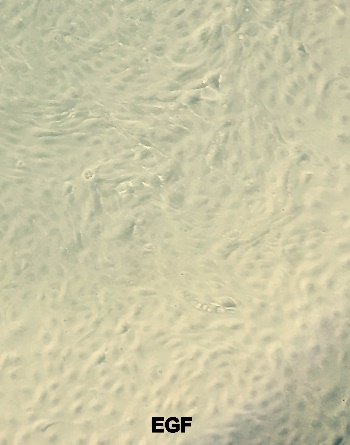

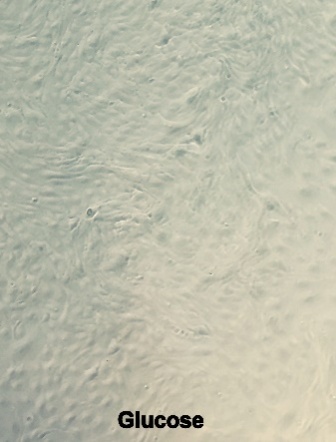

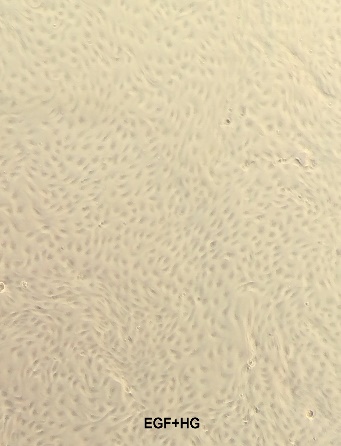


**HG and EGF induced morphological change of MCF-10A and GMECs**. Cells were treated with different doses of HG and EGF 24 h, and the change in morphology was imaged by inverted microscopy.
