# Supplementary figures and images for: Epithelial to mesenchymal transition in mammary gland tissue fibrosis and insights into drug therapeutics
Source: PeerJ. 2023 May 9;11:e15207. doi: 10.7717/peerj.15207 (PMC10178283; doi:10.7717/peerj.15207)

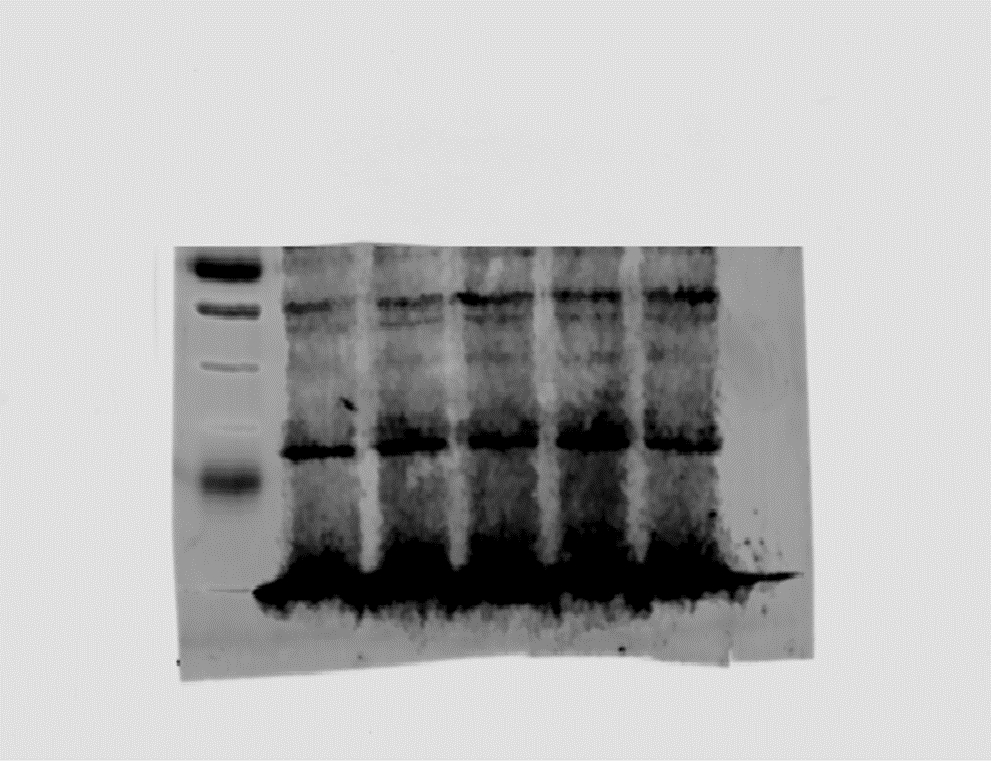

Supplement: Supplemental Information 2 [file peerj-11-15207-s002.zip › Collagen blot.png]

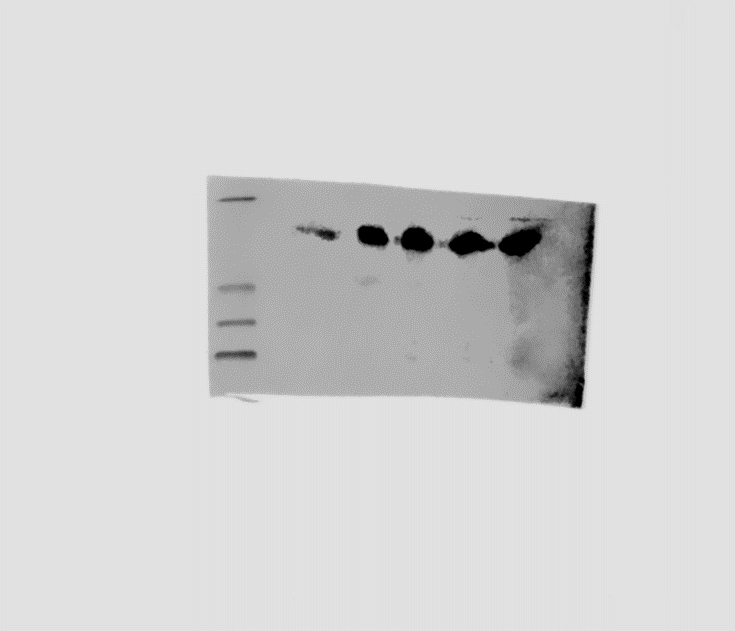

Supplement: Supplemental Information 2 [file peerj-11-15207-s002.zip › Collagen.png]

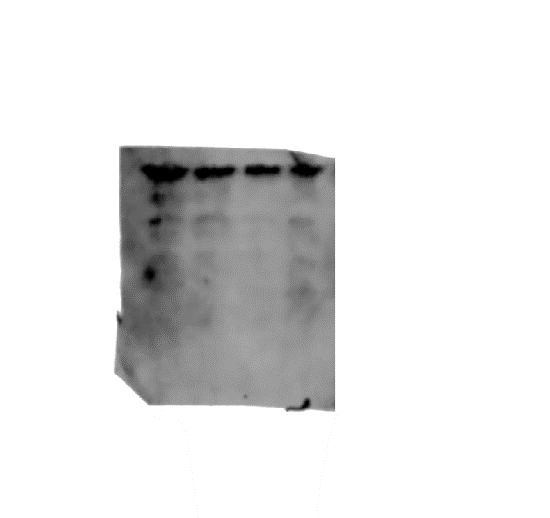

Supplement: Supplemental Information 2 [file peerj-11-15207-s002.zip › collagen1.png]

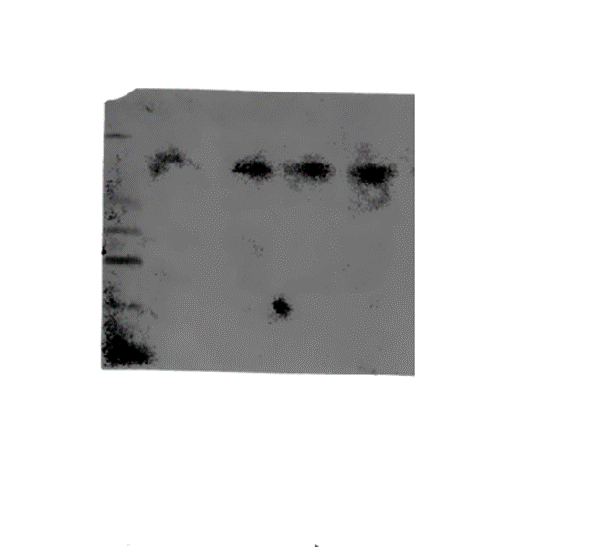

Supplement: Supplemental Information 2 [file peerj-11-15207-s002.zip › collagen2.png]

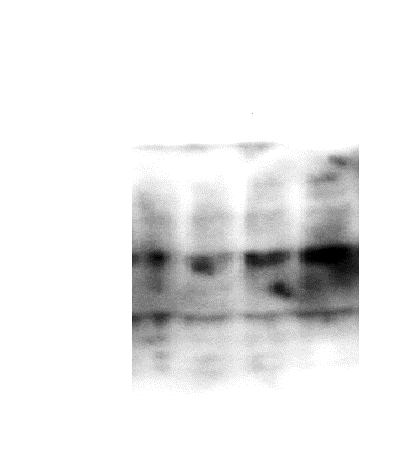

Supplement: Supplemental Information 2 [file peerj-11-15207-s002.zip › Collagen3.png]

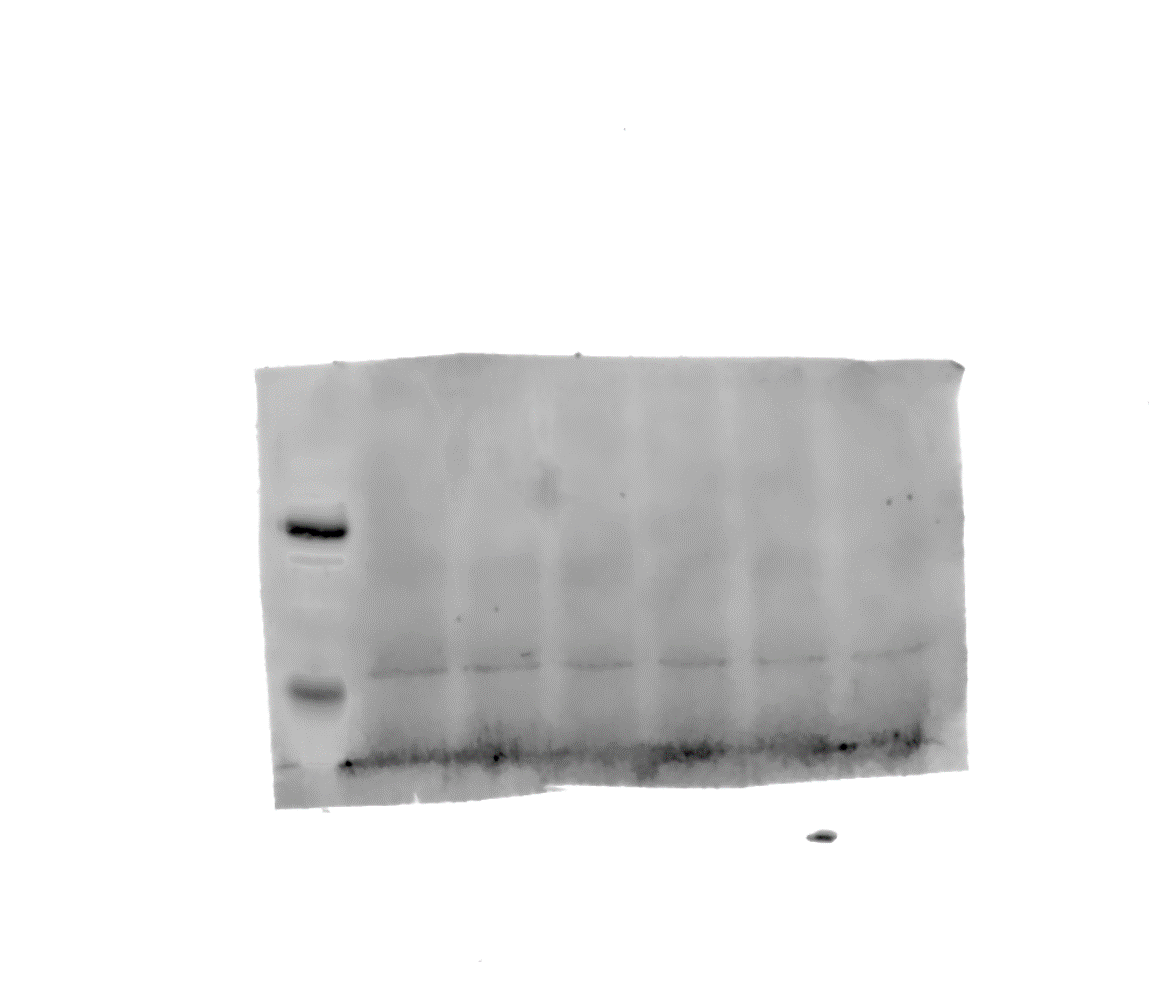

Supplement: Supplemental Information 2 [file peerj-11-15207-s002.zip › loading control.png]

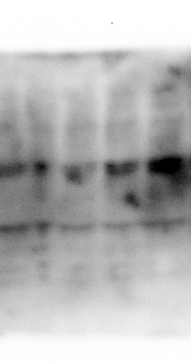

Supplement: Data S1 [file peerj-11-15207-s003.zip › Collagen blot.png]

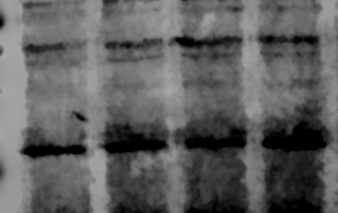

Supplement: Data S1 [file peerj-11-15207-s003.zip › Collagen.png]
